# Detection of *Mycobacterium tuberculosis* Complex and Differentiation of Five Common Nontuberculous Mycobacteria Directly from Clinical Specimens Using a Multiplex PCR Assay

**DOI:** 10.3390/microorganisms14071481

**Published:** 2026-07-07

**Authors:** Keun Ju Kim, Seung Gyu Yun, Yunhee Chang, Myung Hyun Nam, Yunjung Cho

**Affiliations:** 1 Department of Laboratory Medicine, Korea University Anam Hospital, Korea University College of Medicine, Seoul 02841, Republic of Korea; koryun@korea.ac.kr (S.G.Y.); yuret@korea.ac.kr (M.H.N.); eqcho1ku@korea.ac.kr (Y.C.); 2 R&D Innovation Center, Seoul Clinical Laboratories, 13 Heungdeok 1-ro, Giheung-Gu, Yongin-si 16954, Gyeonggi-do, Republic of Korea; yunhee_chang@scllab.co.kr

**Keywords:** *Mycobacterium tuberculosis* complex, nontuberculous mycobacteria, multiplex PCR, line probe assay, melting curve analysis, respiratory specimens

## Abstract

Rapid detection and differentiation of *Mycobacterium tuberculosis* complex (MTBC) and nontuberculous mycobacteria (NTM) are crucial for appropriate treatment selection. The NeoPlex™ TB/NTM Detection Kit is a PCR assay that simultaneously detects MTBC and NTM and identifies five clinically important NTM species (*M. intracellulare*, *M. avium*, *M. kansasii*, *M. abscessus* subsp. *abscessus*, and *M. abscessus* subsp. *massiliense*). We evaluated its comparative analytical performance using 200 stored nucleic acid extracts from respiratory and non-respiratory MTBC- or NTM-positive clinical specimens after excluding 27 specimens without interpretable species-level results. Results were compared with those of a line probe assay (LPA), which formed the reference test. Concordance with the LPA was 93.0%, including 96.9% for MTBC and 92.3% for NTM. The NeoPlex assay correctly differentiated all MTBC- and NTM-positive specimens included in the study cohort. For species-level identification, sensitivity and specificity were 98.9% and 100% for *M. intracellulare*, 97.6% and 100% for *M. avium*, 82.4% and 100% for *M. kansasii*, 79.0% and 100% for *M. abscessus* subsp. *abscessus*, and 100% and 100% for *M. abscessus* subsp. *massiliense*, respectively. These findings suggest that this multiplex PCR assay enables rapid, accurate detection and differentiation of MTBC and NTM species directly from clinical samples.

## 1. Introduction

Tuberculosis (TB), caused by *Mycobacterium tuberculosis* complex (MTBC), remains a major global health challenge despite substantial advances in diagnosis and treatment. According to the World Health Organization, more than 10 million people develop TB annually, and over 1 million deaths are attributed to the disease each year. This makes TB the leading causes of mortality from a single infectious agent worldwide [1].

Nontuberculous mycobacteria (NTM) comprise all species within the genus *Mycobacterium* except members of MTBC and the leprosy-causing species, *M. leprae* and *M. lepromatosis* [2]. These environmental organisms are increasingly recognized as important human pathogens and can cause a broad spectrum of pulmonary and extrapulmonary diseases, particularly in individuals with structural lung abnormalities or impaired immune function [3]. The global burden of NTM disease has increased over recent decades, likely reflecting improved diagnostic capabilities and greater clinical awareness [4,5]. Accurate identification of mycobacteria is critical for patient management because species differ substantially in virulence, antimicrobial susceptibility, and clinical outcomes. Therefore, rapid differentiation of MTBC from NTM and reliable species-level identification of NTM are essential to guide treatment decisions [3,6,7].

*Mycobacterium avium* complex (MAC), including *M. intracellulare* and *M. avium*, is the most common cause of NTM pulmonary disease in many regions worldwide [5]. Higher mortality has been reported among patients infected with *M. intracellulare* than among those infected with *M. avium* [8]. Members of the *M. abscessus* complex, particularly *M. abscessus* subsp. *abscessus* and *M. abscessus* subsp. *massiliense*, require different therapeutic approaches. This is because they differ in the *erm(41)* gene status, which determines inducible macrolide resistance and, consequently, clinical outcomes [9]. *M. kansasii* frequently causes pulmonary disease that closely resembles TB in its clinical presentation and radiographic findings, often resulting in diagnostic challenges [10]. Accordingly, prompt identification of clinically relevant NTM species can facilitate timely treatment selection and improve patient care. In addition, the diagnosis of TB remains challenging because disease development and progression are influenced by complex interactions between the pathogen and host factors, including genetic susceptibility, resulting in substantial heterogeneity in clinical manifestations [11]. This biological heterogeneity can complicate clinical assessment and highlights the continued importance of microbiological confirmation for establishing an accurate diagnosis.

Conventional mycobacterial identification has relied on culture followed by phenotypic and biochemical characterization. However, these approaches are labor-intensive and often require several weeks because many mycobacterial species grow slowly. Specimen processing and culture-based workflows involve multiple manual and repetitive procedures that can increase laboratory workload and occupational exposure risks [12]. Although molecular and proteomic technologies, including gene sequencing, line probe assays (LPAs), and matrix-assisted laser desorption/ionization time-of-flight (MALDI-TOF) mass spectrometry, have substantially improved identification accuracy, they are generally applied only after culture growth has been obtained [13]. Gene sequencing remains the reference method for species identification but is limited by cost, technical complexity, and relatively long turnaround times [14]. LPAs are widely implemented in clinical laboratories but require multiple manual processing steps and experienced personnel [15]. The application of MALDI-TOF mass spectrometry to mycobacteria remains challenging because efficient protein extraction is hindered by the lipid-rich mycobacterial cell wall [16]. These limitations underscore the need for diagnostic approaches that provide accurate species identification while improving laboratory efficiency.

Molecular assays that detect and identify mycobacteria directly from clinical specimens have the potential to shorten diagnostic turnaround times and support earlier therapeutic interventions [17,18]. The NeoPlex™ TB/NTM Detection Kit (GeneMatrix, Seongnam, Republic of Korea) is a multiplex real-time PCR assay that simultaneously detects MTBC and NTM while identifying five clinically important NTM species: *M. intracellulare*, *M. avium*, *M. kansasii*, *M. abscessus* subsp. *abscessus*, and *M. abscessus* subsp. *massiliense*. Although we previously evaluated performance of the NeoPlex assay using cultured mycobacterial isolates [19], validation using cultured isolates does not fully reflect its intended clinical application. In routine practice, mycobacterial culture requires prolonged incubation, and species identification is only possible after successful organism recovery, which may not always be achieved, particularly for fastidious mycobacteria or specimens with a low mycobacterial burden. Consequently, rapid species differentiation directly from clinical specimens has the potential to shorten the diagnostic process, facilitate earlier therapeutic decision-making, and improve patient management. Accordingly, the present study extends our previous findings by evaluating the performance of the NeoPlex assay using nucleic acids extracted directly from clinical specimens and comparing the results with those obtained using an LPA.

## 2. Materials and Methods

### 2.1. Direct Clinical Specimens

This comparative study was conducted at Korea University Anam Hospital between September 2025 and January 2026 using residual nucleic acid extracts obtained from clinical specimens collected between January 2023 and January 2025. Clinical specimens that had previously tested positive for MTBC or NTM by the AdvanSure TB/NTM real-time PCR assay (Invitros, Seoul, Republic of Korea) were eligible for inclusion. Following routine diagnostic testing, the residual nucleic acid extracts were stored at −80 °C until retesting, with storage durations ranging from 8 to 32 months. During the study period, the stored nucleic acid extracts were thawed and newly tested using both the NeoPlex™ TB/NTM Detection Kit and the GenoType Mycobacteria CMdirect Ver. 1.0 assay (Bruker-Hain Diagnostics, Nehren, Germany) for comparative evaluation.

**Figure 1:**
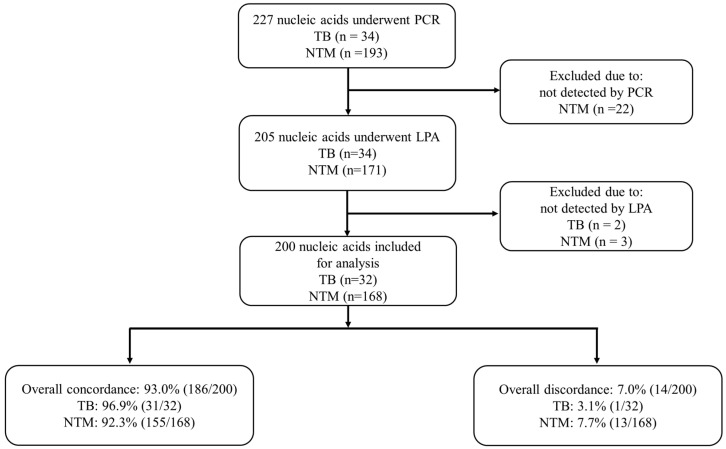
Flow diagram of specimen selection and comparative analysis workflow. Residual nucleic acid extracts were tested using the NeoPlex TB/NTM PCR. PCR-positive specimens were analyzed using the GenoType Mycobacteria CMdirect assay, and concordance and discordance between the two assays were evaluated. Abbreviations: TB, tuberculosis; NTM, nontuberculous mycobacteria; PCR, the NeoPlex™ TB/NTM Detection Kit; LPA (line probe assay), the GenoType Mycobacteria CMdirect assay.

A total of 227 nucleic acid extracts, including 34 MTBC-positive and 193 NTM-positive specimens, were analyzed. Among them, 22 NTM-positive specimens yielded negative results with the NeoPlex assay. Because species differentiation could not be compared in specimens without an interpretable NeoPlex identification result, these specimens were excluded from the comparative analysis. The remaining 205 NeoPlex-positive specimens were subsequently analyzed using the same nucleic acid extracts with the GenoType Mycobacteria CMdirect Ver. 1.0 assay. Five specimens (two MTBC-positive and three NTM-positive specimens) that yielded negative results with the GenoType assay were further excluded because reference species identification was unavailable for comparison. Consequently, 200 specimens, including 32 MTBC-positive and 168 NTM-positive specimens, were included in the final comparative analysis (Figure 1). This study was designed as a comparative performance evaluation using preselected MTBC- or NTM-positive specimens and not as a diagnostic accuracy study in an unselected clinical population.

Among the included specimens, 186 (93.0%) were pulmonary specimens, consisting of bronchial washing specimens (*n* = 138), sputum specimens (*n* = 35), induced sputum specimens (*n* = 10), and tracheobronchial specimens (*n* = 3). The remaining 14 specimens (7.0%) were extrapulmonary specimens, including tissue specimens (*n* = 11) and synovial fluid specimens (*n* = 3) (Table 1).

### 2.2. NeoPlex Assay

The NeoPlex assay was performed as previously described [19]. Briefly, residual nucleic acid extracts from clinical specimens that had been stored at −80 °C after routine diagnostic testing were thawed and used as template DNA. Approximately 5 μL of extract was added to each PCR reaction. The assay is a multiplex real-time PCR assay targeting *IS6110* for MTBC, 16S rRNA for *Mycobacterium* species, and proprietary markers for five clinically important NTM species—*M. intracellulare*, *M. avium*, *M. kansasii*, *M. abscessus* subsp. *abscessus*, and *M. abscessus* subsp. *massiliense*. Species identification is achieved through melting curve analysis following real-time PCR amplification on the CFX96 real-time PCR system (Bio-Rad, Hercules, CA, USA). The assay is intended for detection and species differentiation of mycobacteria and does not assess molecular antimicrobial resistance markers.

### 2.3. LPA

The GenoType Mycobacteria CMdirect Ver. 1.0 assay (Bruker-Hain Diagnostics) was performed according to the manufacturer’s instructions using the same stored nucleic acid extracts used for the NeoPlex assay. Briefly, 5 μL of nucleic acid extract was added to each reaction. The assay is based on DNA-STRIP technology and consists of multiplex PCR amplification using biotinylated primers followed by reverse hybridization to species-specific oligonucleotide probes immobilized on membrane strips. Hybridization signals were visualized by a colorimetric reaction and interpreted according to the corresponding banding patterns. The assay detects MTBC and differentiates several clinically relevant NTM, including *M. avium*, *M. chelonae*, *M. abscessus* complex, *M. fortuitum* group, *M. gordonae*, *M. intracellulare*, *M. scrofulaceum*/*M. intracellulare*, *M. szulgai*, *M. interjectum*, *M. kansasii*, *M. malmoense*, *M. marinum*/*M. ulcerans*, and *M. xenopi*.

### 2.4. Comparison of NeoPlex and GenoType CMdirect Results

The GenoType Mycobacteria CMdirect Ver. 1.0 assay was selected as the reference comparator because it is specifically designed for direct identification and differentiation of clinically relevant mycobacterial species from clinical specimens without prior culture. As both the NeoPlex assay and the GenoType assay are intended for direct testing of clinical specimens, comparison using the same stored nucleic acid extracts enabled evaluation under equivalent analytical conditions. Furthermore, this approach is consistent with the routine diagnostic workflow of the Korea Institute of Tuberculosis (KIT), a WHO Supranational Reference Laboratory, where LPAs are routinely incorporated into the diagnostic workflow for mycobacterial species identification, while sequencing is reserved for specimens with unresolved or ambiguous identification results. In addition, LPAs provide an important practical advantage for evaluating mixed-species specimens because clinically relevant species included in the assay panel can be identified simultaneously by species-specific hybridization probes, whereas sequencing of mixed DNA templates frequently generates overlapping chromatograms that preclude reliable discrimination of individual species. Therefore, the GenoType assay was considered an appropriate reference comparator for evaluating the species differentiation performance of the NeoPlex assay.

Concordance was evaluated by comparing the NeoPlex results with those obtained using the GenoType assay as the reference comparator. NeoPlex results were considered concordant when the assay correctly identified the mycobacterial species included in its detection panel, including cases in which the GenoType assay identified additional species not targeted by the NeoPlex assay. For specimens containing multiple NTM species, concordance was determined according to whether the NeoPlex assay correctly identified the target species included in its detection panel.

The GenoType assay does not differentiate members of *M. abscessus* complex at the subspecies level. Therefore, specimens identified as *M. abscessus* subsp. *abscessus* or *M. abscessus* subsp. *massiliense* by the NeoPlex assay were further characterized by sequencing.

For discordant analysis, residual nucleic acid extracts were sent to the KIT for further characterization. Species identification was established according to the routine diagnostic workflow of the institute based on the combined interpretation of the LPA (Advansure Mycobacteria GenoBlot Assay, Invitros) and sequencing using 16S rRNA, *rpoB*, and *hsp65* targets. The final adjudicated species identification served as the reference standard for discrepancy resolution and subsequent performance analysis.

Sensitivity, specificity, overall agreement, Cohen’s kappa coefficient, and corresponding 95% confidence intervals were calculated. For specimens with concordant results, species identification determined by the GenoType assay was accepted as the reference comparator. For discordant specimens, the final species identification established by the KIT served as the adjudicated reference for discrepancy resolution. Sensitivity and specificity for each NeoPlex target were then calculated on a species-by-species basis using these reference assignments. For mixed-species specimens, each NeoPlex target species was evaluated independently according to the final reference assignment. Accordingly, a specimen could contribute to sensitivity calculations for target species confirmed to be present and to specificity calculations for target species confirmed to be absent. For specificity analysis, specimens confirmed to lack the corresponding target species were considered true-negative specimens, regardless of the presence of other mycobacterial species. Statistical analyses were performed using GraphPad Prism version 11.0.2 for Windows (GraphPad Software, Boston, MA, USA).

## 3. Results

### 3.1. Species Identification Using NeoPlex Assay

Overall, 200 clinical specimens, including 32 MTBC-positive and 168 NTM-positive specimens, were included in the final comparative analysis (Figure 1). Direct comparison between the assays based on the adjudicated species categorization showed a concordance of 93.0% (95% confidence interval [CI], 88.6–95.8) (186/200), with a Cohen’s kappa of 0.73 (95% CI, 0.59–0.86). Concordance was 96.9% (95% CI, 84.3–99.4) (31/32) for MTBC-positive specimens and 92.3% (95% CI 87.2–95.4) (155/168) for NTM-positive specimens. Fourteen specimens (7.0%) yielded discordant results, including one MTBC-positive specimen (3.1%) and 13 NTM-positive specimens (7.7%).

Of the 186 concordant results, 155 were NTM-positive and 31 were MTBC-positive (Table 2). Among the concordant NTM-positive specimens, 133 (85.8%) contained only NeoPlex on-panel species, including *M. intracellulare* (*n* = 69), *M. avium* (*n* = 22), *M. kansasii* (*n* = 10), and *M. abscessus* complex detections (*n* = 17). Co-detection of *M. intracellulare* and *M. avium* was the most common on-panel multispecies combination (*n* = 10). Off-panel NTM species accounted for 16 concordant specimens, including *M. chelonae* (*n* = 7), *M. gordonae* (*n* = 5), and *M. fortuitum* group (*n* = 2), all of which were reported as “NTM” by the NeoPlex assay. Mixed on-/off-panel NTM detections were observed in six specimens. Representative combinations included *M. avium* with *M. chelonae*, *M. kansasii* with *M. chelonae*, and *M. intracellulare* with *M. kansasii* and *M. malmoense*. In these cases, the NeoPlex assay identified the detectable on-panel species, whereas off-panel organisms were not differentiated at the species level. Of the 31 concordant MTBC-positive specimens, 30 contained MTBC alone, whereas one showed simultaneous detection of MTBC with *M. intracellulare* and *M. avium* (Table 2).

A total of 14 discordant results were identified, including 13 NTM and one MTBC specimen (Table 3). The most frequent discordant pattern was failure of the NeoPlex assay to provide species-level identification for on-panel NTM species, resulting in genus-level reporting as “NTM”. This occurred in one *M. intracellulare*, two *M. kansasii*, and four *M. abscessus* complex specimens. Conversely, one specimen was misidentified by the LPA, which reported only NTM, whereas the NeoPlex assay identified *M. avium*. The final reference result supported the NeoPlex identification. Additional species detection by the NeoPlex assay was observed in two specimens, including co-detection of *M. kansasii* with *M. intracellulare* and *M. abscessus* subsp. *massiliense* with *M. intracellulare*. In contrast, the LPA failed to detect additional species in three mixed NTM specimens, all of which were confirmed by the reference laboratory. The discordant MTBC specimen was reported as NTM by the LPA but as MTBC by the NeoPlex assay, which was confirmed by the final reference result. Of the 13 discordant NTM specimens, 10 had Ct values > 35, suggesting a possible low mycobacterial burden.

### 3.2. Sensitivity and Specificity of NeoPlex

The NeoPlex TB/NTM assay correctly differentiated all the MTBC and NTM-positive specimens (Table 4). For species-level identification, the sensitivities ranged from 79.0% for *M. abscessus* subsp. *abscessus* to 100% for *M. abscessus* subsp. *massiliense*. Meanwhile, specificity was 100% for all five target NTM species. The combined sensitivity and specificity for identification of the five target NTM species were 94.8% and 100%, respectively (Table 4).

## 4. Discussion

The rapid and accurate differentiation of MTBC from NTM, along with species-level identification of NTM, is essential for timely therapeutic intervention and appropriate patient management [20]. Here, we evaluated the performance of the NeoPlex TB/NTM PCR assay using nucleic acids extracted from respiratory and non-respiratory clinical specimens. Within this comparative performance evaluation, the assay correctly differentiated all MTBC- and NTM-positive specimens and demonstrated an overall concordance of 93.0% (186/200) with the reference LPA for species-level identification.

Species-level identification plays a critical role in patient management, as treatment strategies and clinical outcomes differ among NTM species [3]. While a previous study evaluated the performance of this PCR assay using cultured isolates [19], direct testing of clinical specimens presents additional challenges. This includes low bacterial burden, specimen heterogeneity, and the presence of PCR inhibitors. Despite these factors, the NeoPlex assay showed a combined sensitivity of 94.8% and a specificity of 100% across the five targeted NTM species (*M. intracellulare*, *M. avium*, *M. kansasii*, *M. abscessus* subsp. *abscessus*, and *M. abscessus* subsp. *massiliense*). These findings support the utility of the assay as a reliable molecular tool for rapid mycobacterial identification directly from patient specimens.

Isolation of NTM and the incidence of NTM pulmonary disease have been increasing globally, primarily driven by MAC [21]. In this context, accurate species-level identification is essential for guiding appropriate therapeutic strategies. Here, the assay had excellent diagnostic performance for MAC, with sensitivities of 98.9% for *M. intracellulare* and 97.6% for *M. avium*. No false-positive results were observed. Given the distinct clinical behavior and disease severity associated with these species, particularly the more aggressive course observed with *M. intracellulare* compared with *M. avium* [8], rapid differentiation may influence clinical decision-making at the early stage of disease evaluation. The assay achieved 100% sensitivity and specificity for *M. abscessus* subsp. *massiliense*. This finding is particularly crucial because *M. abscessus* subsp. *massiliense* generally lacks functional inducible macrolide resistance mediated by the *erm(41)* gene. In addition, it is associated with more favorable treatment responses to macrolide-containing regimens than *M. abscessus* subsp. *abscessus* [9,22,23].

NTM and TB both cause pulmonary and extrapulmonary disease with overlapping clinical presentations, and coinfection has been reported [24]. Although generally infrequent, reported prevalences range from 0.2% to 2.8% among patients with TB [24,25,26] and up to 8% among patients with NTM [27] in selected cohorts. While uncommon, TB/NTM coinfection is likely clinically relevant and may complicate diagnosis. Therefore, laboratory methods that can concurrently detect both MTBC and NTM are of considerable clinical value. Here, the NeoPlex assay was used to identify a specimen containing *M. intracellulare*, *M. avium*, and MTBC concurrently, which demonstrates its ability to detect complex mixed mycobacterial infections. This finding is noteworthy because the two NTM species were not detected previously, which reflects the ability of the assay to identify clinically relevant yet “hidden” NTM, including in the presence of MTBC. In contrast, our previous study demonstrated detection of “hidden” MTBC in a specimen containing the rapidly growing *M. abscessus* subsp. *massiliense* [19]. Taken together, these findings suggest that the NeoPlex assay can detect MTBC–NTM coexistence, supporting its use for simultaneous detection in routine clinical practice.

Analysis of discordant results provided additional insight into the performance of this PCR assay. While the assay demonstrated adequate sensitivity for MAC species, several *M. kansasii* and *M. abscessus* subsp. *abscessus* specimens were identified only at the NTM genus level (Table 3). The underlying cause of these discordant results remains uncertain. One potential explanation is competition between genus-level and species-specific targets within the same multiplex reaction in a single tube. In specimens with limited amounts of mycobacterial DNA, preferential detection of the genus-level target may reduce the sensitivity of downstream species-specific detection. This results in NTM genus positivity without definitive species-level identification. Consistent with this interpretation, 10 of the 13 discordant NTM specimens had high Ct values (>35), suggesting a low concentration of target DNA. Among the 3 discordant *M. kansasii* specimens, 2 had Ct values near the assay detection limit (40 cycles; 39.6 and 39.3). Meanwhile, the remaining specimen had a Ct value of 30.5. Similarly, the four discordant *M. abscessus* subsp. *abscessus* specimens had high Ct values of 37.1, 38.7, 37.2, and 35.8. These findings suggest that low target abundance may have contributed to reduced species-level detection. Owing to the limited numbers of *M. kansasii* (*n* = 10) and *M. abscessus* subsp. *abscessus* (*n* = 14) specimens, a small number of discordant results may have influenced the observed sensitivities. A previous evaluation of the NeoPlex assay using cultured isolates also indicated lower sensitivity for *M. kansasii* than for the other targeted NTM species [19]. Given that *M. kansasii* pulmonary disease frequently mimics tuberculosis clinically and radiologically [10], accurate identification of this pathogen remains particularly important in routine diagnostic practice. Likewise, species-level identification of *M. abscessus* subsp. *abscessus* is clinically important because it can influence subsequent therapeutic decision-making. Therefore, genus-level positivity without species-level identification should be interpreted cautiously, particularly in specimens with low bacterial loads.

**Figure 2:**
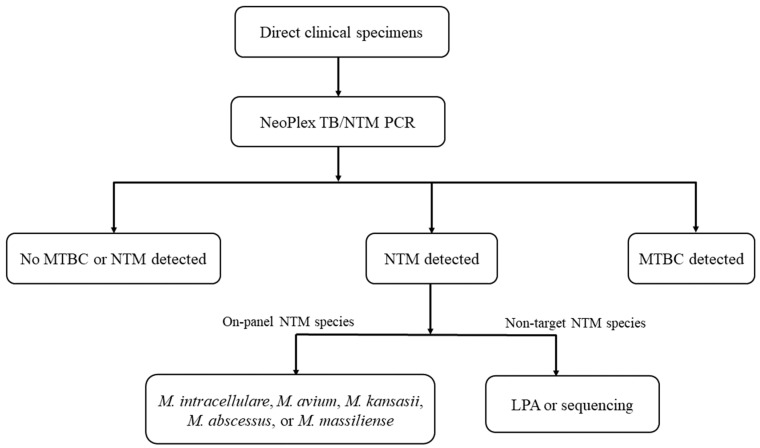
Proposed diagnostic workflow for the NeoPlex TB/NTM PCR assay. Direct specimens are screened by NeoPlex TB/NTM PCR; results are reported as MTBC, target NTM species, or NTM detected without species-level identification, followed by LPA, sequencing, or other reference methods. TB, tuberculosis; MTBC, *Mycobacterium tuberculosis* complex; NTM, nontuberculous mycobacteria; LPA, line probe assay.

Based on these findings, we propose a practical diagnostic workflow for the application of the NeoPlex TB/NTM PCR assay in clinical microbiology laboratories (Figure 2). Specimens can first undergo rapid screening using the multiplex PCR assay. When one of the five targeted NTM species is identified, species-level results can be reported without additional molecular testing. In contrast, specimens with NTM positivity without species-level identification can be referred for additional characterization using LPA, sequencing, or other reference methods. Such an approach may improve laboratory efficiency by providing rapid answers for most clinically relevant cases while reserving additional methods for unresolved or uncommon NTM species.

Beyond the positive performance observed in this study, direct testing of clinical specimens offers several practical advantages in mycobacterial diagnostics. Although mycobacterial culture remains the reference standard for diagnosis, recovery of viable organisms can be influenced by several factors, including prior antimicrobial exposure, low bacterial burden, host-derived antimicrobial peptides, and the presence of viable but non-culturable organisms [28,29,30]. Decontamination procedures routinely performed before mycobacterial culture may reduce recovery of viable mycobacteria, particularly in paucibacillary specimens [31]. Therefore, clinically significant mycobacterial DNA can remain detectable, including when culture results are negative or delayed. In this context, direct molecular detection and identification of MTBC and clinically important NTM species from patient specimens can provide actionable diagnostic information at an earlier stage of the diagnostic process. In this context, the present study highlights the clinical value of direct specimen-based molecular testing, enabling rapid detection and differentiation of MTBC and major NTM pathogens without the need to wait for culture results.

This study has some limitations. First, respiratory specimens accounted for most samples included in this study. Meanwhile, non-respiratory specimens represented a relatively small proportion of the cohort. Therefore, subgroup analysis according to specimen type was not feasible. Nevertheless, the non-respiratory specimens consisted of tissue and synovial fluid collected from normally sterile body sites, where detection of mycobacteria is generally considered clinically significant. Future studies involving a larger and more diverse collection of extrapulmonary specimens are warranted to further validate the assay across different clinical settings. An additional limitation is that the NeoPlex assay targets only five clinically important NTM species. Although these organisms account for a substantial proportion of NTM disease, several off-panel species were identified in this study, including *M. chelonae*, which represents the most common non-target species. *M. chelonae* is a rapidly growing mycobacterium associated with skin and soft tissue infections, catheter-related infections, and occasionally pulmonary disease, particularly in immunocompromised hosts. Therefore, NTM genus-level positivity without species-level identification may require further characterization using LPA, sequencing, or culture-based methods. The study population consisted exclusively of MTBC- or NTM-positive specimens. Consequently, the performance estimates may not be fully generalizable to an unselected clinical population, and the reported sensitivity and specificity should be interpreted in the context of this preselected analytical cohort. Finally, comparison of our findings with those of comparable commercial molecular assays was limited by the lack of published clinical performance data.

## 5. Conclusions

The NeoPlex TB/NTM PCR assay showed high performance for the detection and differentiation of MTBC and NTM from clinical specimens. It showed high concordance with the reference LPA and enabled simultaneous identification of five clinically important nontuberculous mycobacterial species in a single test.

## Figures and Tables

**Table 1:** Direct clinical specimens in the study.

Specimen Site	Specimens	No. (%)
Pulmonary	Bronchial washing	138 (69.0)
	Sputum	35 (17.5)
	Induced sputum	10 (5.0)
	Tracheobronchial	3 (1.5)
	Subtotal	186 (93.0)
Extrapulmonary	Tissue	11 (5.5)
	Synovial fluid	3 (1.5)
	Subtotal	14 (7.0)
Total		200 (100)

**Table 2:** Distribution of concordant results between LPA and the NeoPlex assay for TB/NTM differentiation.

Category	LPA Results	NeoPlex™ TB/NTM Detection Kit	No. of Cases
On-panel NTM species ^a^ (n = 133)	*M. intracellulare*	*M. intracellulare*	69
	*M. avium*	*M. avium*	22
	*M. kansasii*	*M. kansasii*	10
	*M. abscessus* complex	*M. abscessus* subsp. *abscessus*	13
	*M. abscessus* complex	*M. abscessus* subsp. *massiliense*	4
	*M. intracellulare*, *M. avium*	*M. intracellulare*, *M. avium*	10
	*M. intracellulare*, *M. abscessus* complex	*M. intracellulare*, *M. abscessus* subsp. *abscessus*	1
	*M. avium*, *M. kansasii*	*M. avium*, *M. kansasii*	1
	*M. avium*, *M. abscessus* complex	*M. avium*, *M. abscessus* subsp. *massiliense*	2
	*M. intracellulare*, *M. avium*, *M. abscessus* complex	*M. intracellulare*, *M. avium*, *M. abscessus* subsp. *massiliense*	1
Off-panel NTM species ^b^ (n = 16)	*M. chelonae*	NTM	7
	*M. fortuitum* group	NTM	2
	*M. gordonae*	NTM	5
	NTM	NTM	2
Mixed NTM species (on-/off-panel) ^c^ (n = 6)	*M. avium*, *M. chelonae*	*M. avium*	1
	*M. kansasii*, *M. chelonae*	*M. kansasii*	1
	*M. abscessus* complex, *M. fortuitum* group	*M. abscessus* subsp. *abscessus*	1
	*M. intracellulare*, *M. kansasii*, *M. malmoense*	*M. intracellulare*, *M. kansasii*	1
	*M. avium*, *M. gordonae*, *M. fortuitum* group	*M. avium*	1
	*M. chelonae*, *M. gordonae*	NTM	1
MTBC (n = 31)	*M. tuberculosis* complex	*M. tuberculosis* complex	30
	*M. tuberculosis* complex, *M. intracellulare*, *M. avium*	*M. tuberculosis* complex, *M. intracellulare*, *M. avium*	1
Total			186

Abbreviations: LPA, line probe assay (The GenoType Mycobacteria CMdirect); MTBC, *Mycobacterium tuberculosis* complex; NTM, nontuberculous mycobacteria. ^a^ On-panel NTM species represent species specifically targeted by the NeoPlex™ TB/NTM Detection Kit (*M. intracellulare*, *M. avium*, *M. kansasii*, *M. abscessus* subsp. *abscessus*, and *M. abscessus* subsp. *massiliense*). Results were considered concordant when the NeoPlex assay identified the same panel-target species as the reference LPA. ^b^ Off-panel NTM species represent species not included in the NeoPlex detection panel. Accordingly, these results were considered concordant when the NeoPlex assay correctly classified these organisms as NTM without assigning an incorrect panel-target species. ^c^ Mixed NTM species (on-/off-panel) refer to specimens containing either both on-panel and off-panel species or off-panel species alone. In specimens containing both, NTM is detected using universal 16S rRNA primers, while species identification is limited to five on-panel species using species-specific primers; off-panel organisms are not differentiated at species level and only on-panel species are reported. In specimens containing only off-panel species (including multiple species), results are reported as NTM without species-level identification. Concordance was defined as correct detection of all NeoPlex species identified by LPA regardless of additional off-panel species.

**Table 3:** Summary of discrepant results between LPA, NeoPlex assay, and adjudicated reference results.

LPA Result	NeoPlex™ TB/NTM Detection Kit	Ct	Adjudicated Result ^a^
Misidentification results using the NeoPlex assay			
*M. intracellulare*	NTM	37.4	*M. intracellulare*
*M. kansasii*	NTM	39.6	*M. kansasii*
*M. kansasii*	NTM	30.5	*M. kansasii*
*M. abscessus* complex	NTM	37.1	*M. abscessus* subsp. *abscessus*
*M. abscessus* complex	NTM	38.7	*M. abscessus* subsp. *abscessus*
*M. abscessus* complex	NTM	37.2	*M. abscessus* subsp. *abscessus*
*M. abscessus* complex	NTM	35.8	*M. abscessus* subsp. *abscessus*
Misidentification results using the LPA			
NTM	*M. avium*	37.6	*M. avium*
Additional detection using the Neoplex assay			
*M. kansasii*	*M. kansasii, M. intracellulare*	34.6	*M. kansasii*, *M. intracellulare*
*M. abscessus* complex	*M. abscessus* subsp. *massiliense*, *M. intracellulare*	26.3	*M. abscessus* subsp. *massiliense*, *M. intracellulare*
Additional detection using the LPA			
*M. intracellulare*, *M. avium*	*M. avium*	38.7	*M. intracellulare*, *M. avium*
*M. intracellulare*, *M. avium*	*M. intracellulare*	38.7	*M. intracellulare*, *M. avium*
*M. intracellulare*, *M. kansasii*	*M. intracellulare*	39.3	*M. intracellulare*, *M. kansasii*
MTBC discordance			
NTM	MTBC	33.7	MTBC

Abbreviations: LPA, line probe assay (The GenoType Mycobacteria CMdirect); Ct, cycle threshold; NTM, nontuberculous mycobacteria; MTBC, *Mycobacterium tuberculosis* complex. ^a^ Adjudicated results were determined at the Korea Institute of Tuberculosis, a WHO Supranational Reference Laboratory, by sending discrepant specimens to this reference center, where the Advansure Mycobacteria GenoBlot LPA (Invitros, Seoul, Republic of Korea) served as the primary method for final interpretation and Sanger sequencing was performed for confirmation when required.

**Table 4:** Diagnostic performance of the NeoPlex TB/NTM assay for TB/NTM detection and differentiation of five major NTM species.

Target	TP	TN	FN	FP	Sensitivity % (95% CI)	Specificity % (95% CI)
*M. intracellulare*	87	111	1	0	98.9 (93.8, 99.9)	100 (96.7, 100)
*M. avium*	41	157	1	0	97.6 (87.7, 99.9)	100 (97.6, 100)
*M. kansasii*	14	182	3	0	82.4 (59.0, 93.8)	100 (97.9, 100)
*M. abscessus* subsp. *abscessus*	15	180	4	0	79.0 (56.7, 91.5)	100 (97.9, 100)
*M. abscessus* subsp. *massiliense*	8	191	0	0	100 (67.6, 100)	100 (98.0, 100)
Combined five species	165	821	9	0	94.8 (90.5, 97.3)	100 (99.5, 100)
NTM	168	31	0	0	100 (97.8, 100)	100 (89.0, 100)
MTBC	32	167	0	0	100 (89.3, 100)	100 (97.8, 100)

Abbreviations: CI, confidence interval; FN, false negative; FP, false positive; MTBC, *Mycobacterium tuberculosis* complex; NTM, nontuberculous mycobacteria; TN, true negative; TP, true positive.

## Data Availability

The original contributions presented in this study are included in the article. Further inquiries can be directed to the corresponding author.
